# Soluble Carbohydrates in Several Transylvanian Potato Cultivars

**DOI:** 10.3390/plants12010070

**Published:** 2022-12-23

**Authors:** Edward Muntean, Nina Bărăscu

**Affiliations:** 1 Department of Food Science, Faculty of Food Science and Technology, University of Agricultural Sciences and Veterinary Medicine Cluj Napoca, 3-5 Calea Manaştur, 400372 Cluj Napoca, Romania; 2 National Institute for Research and Development for Potato and Sugar Beet Brasov, 2 Fundăturii Str., 500470 Brașov, Romania

**Keywords:** potatoes, fructose, glucose, sucrose, carbohydrates, HPLC, acrylamide precursors

## Abstract

This paper is the first to report the soluble carbohydrate content at harvest for eight Transylvanian potato cultivars: Christian, Cumidava, Kronstadt, Riviera, Roclas, Rustic, Tampa and Zamolxis. The aim of this study is to explore the soluble carbohydrate composition of the above-mentioned cultivars, since such quantitative information is important for breeding programs, consumers and processing units. High performance liquid chromatography was used for analysis, separations being achieved using a Prominence Shimadzu system with a refractive index detector, under isocratic conditions with a mobile phase consisting of acetonitrile: water (80:20%) delivered at 1 mL/min; baseline separations of the target analytes were accomplished with an EC 250/4 Nucleodur 100–5 NH_2_ RP column in less than 10 min. The carbohydrate concentrations were found to range from 24.03 mg/100 g (Zamolxis) to 76.58 mg/100 g (Riviera) for fructose, while the corresponding range was from 52.78 mg/100 g (Zamolxis) to 232.97 mg/100 g (Riviera) for glucose and from 238.41 mg/100 g (Zamolxis) to 378.45 (Cumidava) for sucrose. Chromatographic data were then subjected to chemometric analysis; the association of these complementary techniques allowed a fast selection of cultivars with low-reducing carbohydrate content for food processing purposes—the cultivars Zamolxis, Kronstadt, Christian and Roclas were outlined exhibiting both the lowest reducing carbohydrate content and the lowest sucrose content.

## 1. Introduction

Potato (*Solanum tuberosum* L.) is the most important vegetable in the human diet; it has a high yielding, a high nutritive value, while giving elevated returns to farmers [1]. Potato is a nutritious, tasty and inexpensive vegetable, a staple food of many people, covering a large share in the economic balance of numerous countries. Potato tubers supply high amounts of carbohydrates and also a significant content of proteins, minerals, carotenoids and vitamin C [2,3,4,5]; they have a wide variety of table, processed, livestock feed and industrial uses, and in the meantime, are much appreciated for their health-related benefits [6,7]. As there is a continuous increase in the demand of processed food, the potato processing industry is a fast growing sector.

The content of soluble carbohydrates from potato is an important factor for both consumers’ acceptability and processing plants, strongly influencing their taste, nutritive value and behavior during the heat treatment; the monosaccharides fructose and glucose (reducing carbohydrates) and the non-reducing disaccharide sucrose are the major soluble carbohydrates found in potato tubers [8,9,10,11,12,13]. The overall soluble carbohydrate content (SCC) of potato tubers is low, at around 0.5–1% of the fresh weight, depending on the factors such as genotype, maturity, physiological state, temperature during growth, mineral nutrition, irrigation, storage duration and storage conditions [10,14,15,16]. During low temperature storage, the SCC increases significantly in most genotypes [17,18,19]; this process, also known as “cold induced sweetening”, depends on factors such as the storage temperature, dormancy break, sprouting after dormancy break and tuber senescence [17].

The reducing carbohydrate content is one of the most important parameters that influences the processing quality of potatoes; a higher value for this renders potato tubers unsuitable for use as raw material for processing, especially for dehydrated and fried products. Potato tubers that contain high concentrations of reducing carbohydrates lead to unacceptable brown colored chips and fries as a result of the Maillard reaction between these compounds and amino acids [20,21]; besides, the Maillard reaction is also related to the formation of the hazardous acrylamide in the case of high-temperature processed potato products [22,23,24,25]. Acrylamide is a reported carcinogen [26], food processing units preferring potato cultivars with lower SCC for limiting its formation [23,27,28,29]. The SCC of potato tubers is important not only for the above-mentioned issues but also for their acceptance by consumers; the higher this content is, the sweeter their taste is. Previous research on SCC from potato tubers revealed that sucrose is the major carbohydrate component of these and the most important involved in their sweetness [8,9,13,15,25,30,31,32].

Among the reported analytical methods utilized for SCC’s determination, the chromatographic methods are the most common techniques [33,34]. The gas chromatographic methods were the first ones able to provide a good resolution for these compounds, but they required a laborious and time-consuming derivatization step [35,36]. High performance liquid chromatography (HPLC) is more convenient regarding sample preparation, currently being the most used method [8,13,37]. HPLC with refractive index detection (RID) was used for the determination of soluble carbohydrates in food products, because of its numerous advantages (e.g., reliability, simplicity, price) [10,32,38,39]. Some major drawbacks of RID such as the lack of sensitivity and the incompatibility with gradients can be compensated using other types of detection, such as evaporative light scattering detection [40,41,42], electrochemical detection [43,44] or mass spectrometry [29,45,46], but prices and systems’ complexity are higher. Using HPLC, non-derivatized carbohydrates are usually separated on silica-based amino columns [47]; ion chromatography can provide an alternative to such separations [29,48,49,50], while capillary electrophoresis offers a different approach [51,52]. In most cases, the soluble carbohydrate extraction was accomplished using ethanolic solutions, heating and mixing providing a higher efficiency for the extraction procedure. Unfortunately, the liquid–solid extraction process is not selective, leading to an extract which contains, besides the desired analytes, many other compounds; some of them can affect the chromatographic separations, leading even to an early degradation of HPLC columns’ performances, hence a sample preparation stage is a must in analysis (Carrez method, dialysis, solid phase extraction-SPE, etc.). SPE is a rapid and reproducible sample preparation technique that allows a selective removal of co-extracted substances [53]. A faster alternative to traditional methods, mainly used in food quality testing, is near infrared spectroscopy, which is able to predict the carbohydrate content in potatoes in minutes, with minimal or no sample preparation [54,55,56,57,58,59].

The purpose of this study is to explore the soluble carbohydrate composition of several Transylvanian potato cultivars, on which, up to the present, there are no data; such quantitative information is important for breeding programs [60], consumers and processing units. The study is relevant because besides dry matter, the SCC is an important quality parameter for assessing the potential of potato tubers to produce acceptable processed products with appropriate color and taste; the sucrose concentration of tubers is also an indicator of their maturity; hence, it can also be considered as a decision tool for harvesting [61]. An optimized HPLC–RID method was used in this study, after an SPE stage, which is necessary since certain matrix compounds can interfere with the chromatographic separation while negatively affecting the analytical column. For a fast highlight of the cultivars with low reducing carbohydrate content which can be considered for food processing purposes, chromatographic data were subjected to chemometric analysis.

## 2. Results

**Figure 1:**
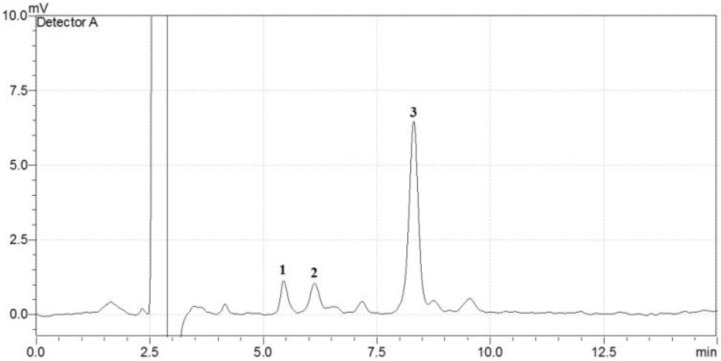
Representative chromatographic fingerprint of soluble carbohydrates having sucrose as major component (Zamolxis cultivar). Peak D’s: 1—fructose, 2—glucose, 3—sucrose.

The SCC of potato tubers is a result of a complex equilibrium between starch degradation, starch biosynthesis and respiration. Chromatographic analysis revealed different soluble carbohydrate fingerprints for the studied genotypes, a representative one being presented in Figure 1.

Table 1 summarizes the obtained data for the studied potato cultivars; it highlights that the major soluble carbohydrate is sucrose, followed by glucose and fructose; for sucrose, the concentration range was from 238.41 mg/100 g (Zamolxis) to 378.45 (Cumidava), for glucose, the corresponding range was from 52.78 mg/100 g (Zamolxis) to 232.97 mg/100 g (Riviera), while for fructose, the concentrations ranged from 24.03 mg/100 g (Zamolxis) to 76.58 mg/100 g (Riviera). The dry matter ranged from 18.74% (Riviera) to 24.11% (Rustic). The lowest concentrations of reducing carbohydrates were recorded for Zamolxis, Kronstadt, Christian and Roclas cultivars (76.81 to 99.01 mg/100 g).

For the fructose content, the obtained results showed no significant differences for the cultivars Christian, Cumidava, Kronstadt, Roclas, Tampa and Zamolxis; however, there was a significant differences (*p* < 0.05) between these and the cultivars Rustic and Riviera. For the glucose content, there were no significant differences for the cultivars Christian, Kronstadt, Roclas and Zamolxis, as well as for the cultivars Cumidava and Tampa/Rustic and Riviera. For sucrose, there were no significant differences for the cultivars Christian, Kronstadt, Riviera, Rustic, Roclas and Zamolxis, as well as for cultivars Cumidava, Rustic and Tampa. Statistical analysis of dry matter contents highlighted only a significant difference, for the cultivar Riviera; for the other cultivars, there were no significant differences (*p* < 0.05). For the most relevant parameter—the reducing carbohydrates—there were no significant differences between cultivars Christian, Kronstadt, Roclas and Zamolxis, as well as for cultivars Cumidava and Tampa; however, there was a significant difference (*p* < 0.05) between these and the cultivars Rustic and Riviera.

**Figure 2:**
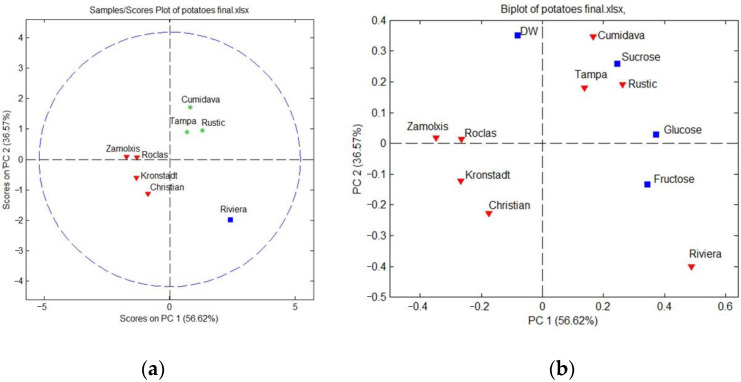
PCA scores (**a**) and biplot (**b**) for potato samples.

Principal component analysis (PCA) of the obtained data was accomplished using four variables (the contents of fructose, glucose and sucrose, as well as the dry matter), leading to the model from Figure 2a, which explains 93.19% of data variability, revealing two data clusters—one including the cultivars Cumidava, Tampa and Rustic, while the other one includes the cultivars Christian, Kronstadt, Roclas and Zamolxis. The first cluster contains medium-late cultivars, suitable for autumn-winter consumption, their tubers being characterized by the highest concentrations of sucrose and glucose and the highest loadings on the second principal component (PC2). The second cluster includes cultivars with lower concentrations of fructose and glucose, with low loadings on both PC1 and PC2. The Riviera cultivar has a distinct position in this context, being the only genotype which exhibited the highest amounts of fructose and glucose, containing in the meantime an important amount of saccharose.

**Figure 3:**
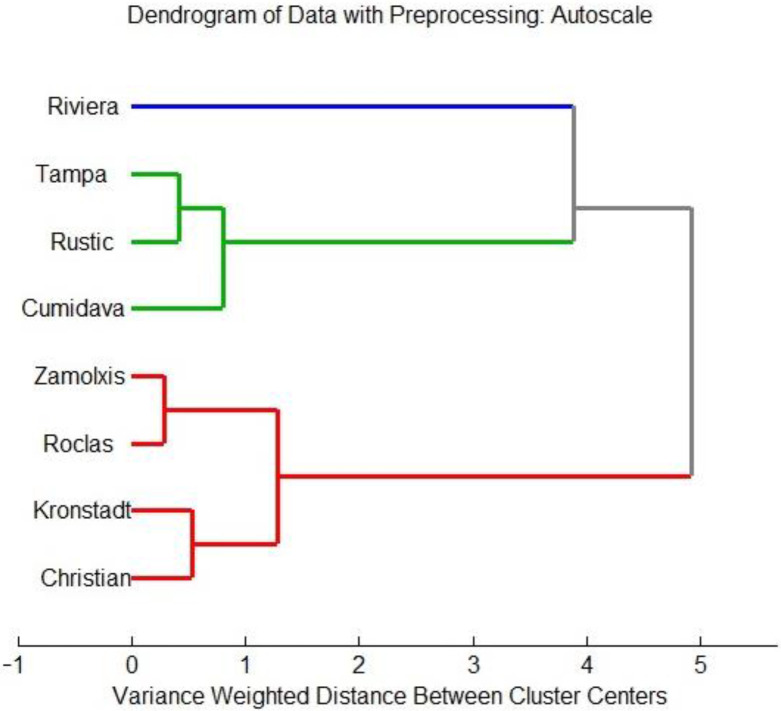
Dendrogram for cluster analysis (Ward’s method, using Mahalanobis distance).

The biplot from Figure 2b reveals a positive correlation between the content of reducing carbohydrates; the correlation analysis (Table 2) for the obtained data also shows a strong and significant correlation between the concentrations of fructose and that of glucose, with a high Pearson’s coefficient (0.8748), which is in agreement with the former reported results [8,12]; sucrose was also significantly correlated with the reducing carbohydrate content but with lower coefficients, showing a moderate relationship with the reducing carbohydrate content for the studied genotypes at harvest. Cluster analysis was accomplished by Ward’s method, using Mahalanobis distance, leading to the dendrogram from Figure 3, validating the results obtained by PCA.

## 3. Discussion

As highlighted in the introductory section, it is generally recognized that the higher the levels of reducing carbohydrates, the lower the suitability of potato tubers for processing is; besides, by the reaction of reducing carbohydrates with free amino groups during frying and baking (Maillard reaction), the formation of a color can make crisps and chips unacceptable for consumers and even hazardous due to the formation of acrylamide. Because an important desirable characteristic of potato genotypes designed for chipping is a low SCC, many researches have been devoted to the investigation of this issue. In similar studies with the reported one various concentrations of soluble carbohydrates were published, but relatively close with ours; comparing our results with most of the available results in the literature, the variability of the SCC in Transylvanian potato genotypes is lower. Hence, three American cultivars were stated with 80–800 mg/100 g fresh weight (FW) fructose, 160–890 mg/100 g FW glucose and 70–260 mg /100 g FW sucrose [13]; four potato cultivars from Cyprus were found to contain 0–27 mg/100 g FW fructose, 0–153 mg/100 g FW glucose and 750–1370 mg /100 g FW sucrose [12]. In a study on nine Italian potato genotypes and 22 American ones, fructose ranged from 0 to 60 mg/100 g FW, glucose from 20 to 620 mg/100 g FW and sucrose from 40 to 1390 mg/100 g FW [25].

Since several authors reported values relative to the dry weight (DW), Table 3 provides another instance of data from Table 2 in which the results were expressed in g/100 g DW. Four potato genotypes from Virginia were found to contain 0.04–0.07 g/100 g DW fructose, 0.06–0.35 g/100 g DW glucose and 0.34–0.66 g/100 g DW sucrose [11]. In studies performed on Colombian genotypes, the authors found a high variability of concentrations, with the following ranges: 0.1–1.48 g/100 g DW fructose, 0.25–4.53 g/100 g DW glucose and 0.93–1.1 g /100 g DW sucrose [9], then 0.03–2.72 g/100 g DW fructose, 0.05–2.80 g/100 g DW glucose and 0.64–2.95 g /100 g DW sucrose [8].

For the fructose content, the obtained results showed no significant differences for the cultivars Christian, Cumidava, Kronstadt, Roclas, Tampa and Zamolxis; however, there is significant difference (*p* < 0.05) between these and the cultivars Rustic and Riviera. For the glucose content, there are no significant differences for the cultivars Christian, Kronstadt, Riviera, Roclas and Zamolxis, as well as for the cultivars Cumidava, Rustic and Tampa. For sucrose, there are no significant differences for the cultivars Christian, Kronstadt, and Rustic, as well as for cultivars Cumidava, Riviera, Rustic and Tampa, respectively for the cultivars Kronstadt and Rustic. For the reducing carbohydrates and for the total SCC, there are no significant differences between cultivars Christian, Kronstadt, Roclas and Zamolxis, as well as for cultivars Cumidava, Rustic and Tampa; however, there is significant difference (*p* < 0.05) between these cultivars and Riviera.

In all published cases, sucrose was the major carbohydrate, while fructose and glucose concentrations were smaller than those of sucrose; this is also the case in our study, with glucose/fructose ratios higher than one, possibly due to the relatively high activity of fructokinase diminishing the content of fructose in tubers [62]. A higher content of glucose than that of fructose is also common in other types of potato at harvest [63,64]. The cultivar with the highest overall SCC (593.26 mg/100 g) and also with the highest reducing carbohydrate content (309.55 mg/100 g) is Riviera, while at the other extreme of the dataset, the Zamolxis cultivar had the lowest overall SCC (315.22 mg/100 g) and the lowest reducing carbohydrate content (76.81 mg/100 g). The recommended values for the reducing carbohydrate content considered to be appropriate for acceptable flavor, browning and acrylamide content in roasted and baked potato products are in the range 0.1–0.5 g/kg DW [65], but in some cases, can be lower than 0.035 g/100 g DW [28].

## 4. Materials and Methods

### 4.1. Plant Material

**Figure 4:**
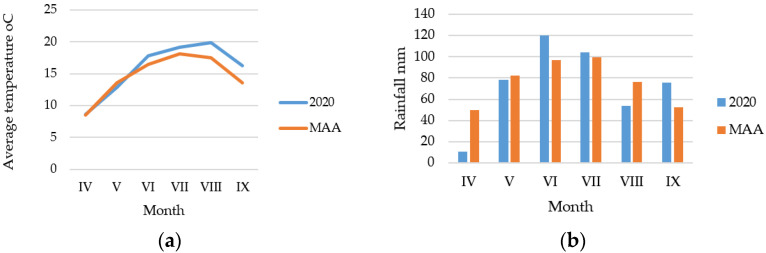
Average temperatures (**a**) and the amount of rainfall (**b**) during the vegetation period (MAA: multi-annual average).

Eight potato varieties (Christian, Cumidava, Kronstadt, Riviera, Roclas, Rustic, Tampa and Zamolxis) were cultivated on the experimental fields of the National Institute of Research and Development for Potato and Sugar Beet Brasov, Romania, under the same growing conditions, on a chernozemic soil with the following characteristics: clay—30%, pH—6, humus—4.5%, mobile phosphorus—50 ppm, mobile potassium—over 100, with the degree of base saturation—85%. The experimental field was located at 45°40′26″ N 25°32′11″ E, in a temperate climate area, with the average temperatures and the amount of rainfall during the vegetation period presented in Figure 4. For basic fertilization, 500 kg/ha complex N:P:K—15:15:15 were applied. The revitalization work was carried out mechanically on 4 May 2020, followed by a pre-emergent herbicide treatment with Surdone (1.2 kg/ha. The emergence of potatoes was delayed due to the low amount of precipitation in April. The climatic conditions imposed the application between 4 June and 20 August of nine treatments for the control of potato blight (*Phitophtora infestans*): 1—Ridomil 2.5 kg/ha; 2—Consento 2.0 L/ha; 3—Lieto 450 g/ha; 4—Ridomil 2.5 kg/ha; 5—Carial Star 0.6 L/ha; 6—Cymco 2.5 kg/ha; 7—Infinito 1.4 L/ha + Lebosol P 5 L/ha; 8—Lieto 450 g/ha; 9—Shirlan 0.4 L/ha. To control aphids and *Leptinotarsa decemlineata*, two treatments were applied, one with Biscaya 0.2 L/ha, the other with Proteus 110OD 0.4 L/ha.

**Figure 5:**
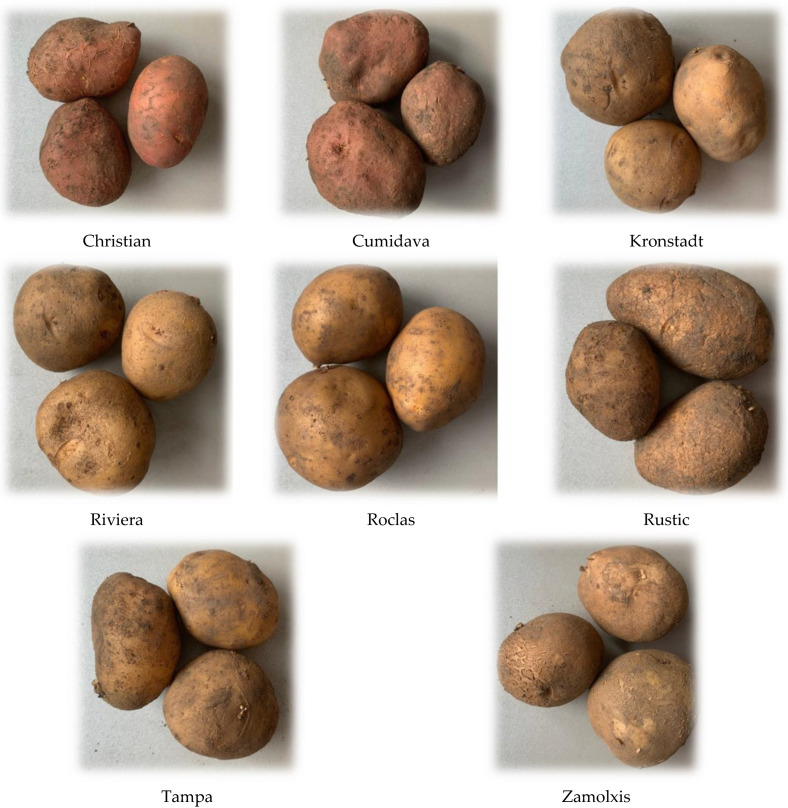
Potato cultivars used in this study.

All cultivars were sown on 19 April 2020, then the tubers were harvested on 2 October for the Riviera cultivar, on 12 October for the Christian, Kronstad, Roclas, Zamolxis cultivars and on 26 October for the Cumidava, Rustic and Tampa cultivars, at the maturity stage (Figure 5). The studied potato varieties fall into the following maturity groups: Riviera is a very early variety, Roclas, Christian, Zamolxis, Kronstad are medium early varieties while Cumidava, Tâmpa and Rustic are medium late varieties. We must mention here that the climatic conditions in the Brasov area do not always ensure the thermohydric needs of the early and semi-early varieties to show their characteristic earliness. Cumidava, Tâmpa and Rustic varieties are suitable for autumn-winter consumption, while the varieties Roclas, Christian, Kronstad are suitable for early and summer–autumn consumption and Zamolxis variety for summer–autumn consumption [66]. The varieties Tâmpa, Roclas, Zamolxis and Kronstad are the most appropriate for processing [67,68]. Most of these potato varieties were patented by the above-mentioned institute [69], being also recorded in the Official Catalogue of Romanian Potato varieties [70].

### 4.2. Chemicals and Standards

HPLC grade acetonitrile and analytical grade ethanol, as well as carbohydrate standards (D-glucose, fructose and sucrose) were from Merck (Darmstadt, Germany). Ultrapure water with a specific resistance of 18.2 ΜΩ. cm^−1^ was utilized for preparation of mobile phases as well as for samples’ dilution, being obtained from a Direct Q 3UV Smart system (Millipore, Darmstadt, Germany). The mobile phase was filtered through a 0.45 μm membrane (Millipore Corp., Bedford, MA, USA) and then degassed using an Elmasonic S30 H ultrasonic bath (Elma Hans Schmidbauer GmbH & Co., Singen, Germany).

### 4.3. Sampling and Extraction

The potato tubers were manually harvested. Five mature and healthy potato tubers without mechanical damages were selected from each cultivar and stored in the dark, at 18 °C, for one week. Prior to analysis, they were washed with tap water and dried with paper towels, then peeled manually and diced. After homogenization, representative samples of around 10 g were selected; these were weighed and subjected to extraction and dry matter determination. Three sample replicates were provided for each cultivar and for each type of determination. Carbohydrate extraction was accomplished in a blender, adding 100 mL of 80% ethanol to each sample and homogenizing it for 2 min; the resulting suspension was transferred in a 250 mL flask which was placed in an ultrasonic bath (Elmasonic S30 H, Elma Hans Schmidbauer GmbH & Co., Germany), where it was kept at 60 °C for one hour. After cooling, 80% ethanol was added to bring the total volume to 250 mL, the system being therefore mixed for a proper homogenization. After sedimentation, 5 mL of the supernatant was collected and subjected to solid phase extraction.

### 4.4. Solid Phase Extraction (SPE)

SPE was used for removing the unwanted co-extracted compounds as well as for protecting the analytical column, using Sep-Pak C_18_ cartridges (Waters Assiciates Inc., Milford, MA, USA). The cartridges were first preconditioned by flushing with 4 mL methanol, then with 4 mL ultrapure water at a flow rate of 1 mL/min [47,53]; 5 mL extracts were passed through the activated cartridges, discarding the first milliliter, then the eluates were collected in 2 mL vials, being subjected to HPLC.

### 4.5. HPLC Analysis

Analyses were performed on a Shimadzu Prominence HPLC system, consisting of one LC-20AP solvent delivery module, a DGU 20As online degasser, an automatic sample injector SIL-10AF, a RID-10A differential refractive index detector, a CTO-20A column oven and a CBM-20A system controller. Isocratic separations were conducted at 35 °C using an EC 250/4 Nucleodur 100–5 NH_2_ RP column (250 × 4.6 mm, Macherey-Nagel)—a multimode column protected by a pre-column (30 mm × 4.6 mm), with a flow rate of 1 mL. Min^−1^ acetonitrile in water (80:20 *v*/*v*%) as the mobile phase—an optimized Macherey-Nagel procedure [71]; the injection volume was 10 μL. Under these conditions, baseline separations of the target carbohydrates were accomplished in less than 10 min. The compounds’ identification was based both on comparison of the retention times with those of standards and on co-elution after spiking with authentic standards. Quantification was performed by the external standard method. Calibration curves were established using a mixture of fructose, glucose and sucrose standards, at five concentrations (Table 4). Recoveries were calculated by analyzing spiked samples, revealing percentages in the range of 95.27% for fructose and 98.83% for sucrose.

### 4.6. The Dry Matter Content

The dry matter content was determined using an AL01-05-100 forced air drying oven (Advantage Lab GmbH, Darmstadt, Germany), by heating samples of around 200 g at 105 °C for eight hours; weighing was accomplished using a Kern ABJ 220 N analytical balance (Kern & Sohn GmbH, Balingen, Germany).

### 4.7. Data Analysis

HPLC instrument control, data acquisition and chromatographic data analysis were accomplished using LCSolution (Shimadzu Corporation, Japan); statistical analysis was performed in Excel (Microsoft) and in SPSS version 17 (SPSS Inc., Chicago, IL, USA), where the means were processed using analysis of variance and Tukey’s honestly significant difference test, at *p* ≤ 0.05. The results were reported as means ± standard error; differences among means are presented using different letters (results with the same letter were not significantly different, at *p* < 0.05). Principal component analysis and cluster analysis were performed using MatLab (Mathworks Inc., Natick, MA, USA) after mean center preprocessing.

## 5. Conclusions

The obtained results may enable consumers and processors to select the most appropriate potato cultivars with low levels of acrylamide precursors for baking or frying, and also for best chipping performances. The association of HPLC and multivariate analysis allows a fast selection of cultivars with low-reducing carbohydrate content, such as Zamolxis, Kronstadt, Christian and Roclas, these also exhibiting the lowest sucrose content (these cultivars will generate the smallest amounts of acrylamide during frying and baking, as well as a desirable color); more than that, this association revealed clusters with biological relevance, providing valuable data for breeding programs.

The analytical approach used here, involving HPLC analysis of carbohydrates employing SPE, provides a rapid and reliable means to determine the SCC in potato tubers with rapid, specific, reliable and sensitive measurements. SPE affords a convenient removal of the matrix interferences, while maintaining the chromatographic column’s integrity and assuring reproducible and good quality separations; it uses small sample and solvent volumes, is fast, simple and relatively cheap, leading to an eluent which can be injected into the HPLC system.

The results of this work contribute relevant knowledge to the SCC of several Transylvanian potato cultivars, enabling a better understanding of the biochemical processes of carbohydrate formation in potato tubers, as well as to use them accordingly as suitable raw materials for processing.

## Figures and Tables

**Table 1:** SCC in potato cultivars at harvest (mean values of three replicates ± standard deviation; means followed by different superscript letters within the same column indicate significant differences, established using one-way ANOVA with post-hoc Tukey’s HSD test (*p* < 0.05)).

Cultivar	Fructose [mg/100 g]		Glucose [mg/100 g]		Sucrose [mg/100 g]		Dry Matter (DM) [%]		Reducing Carbohydrates [mg/100 g]
Christian	35.12	±1.73 ^c^	60.58	±2.96 ^c^	260.12	±13.04 ^b^	20.20	±1.20 ^a^	95.70 ^d^
Cumidava	38.91	±1.68 ^c^	154.51	±7.61 ^b^	378.45	±18.91 ^a^	24.05	±1.43 ^a^	193.42 ^c^
Kronstadt	25.60	±1.21 ^c^	63.83	±2.87 ^c^	249.83	±12.47 ^b^	21.40	±1.29 ^a^	89.43 ^d^
Riviera	76.58	±3.75 ^a^	232.97	±11.45 ^a^	283.71	±14.11 ^b^	18.74	±1.05 ^b^	309.55 ^a^
Roclas	25.43	±1.25 ^c^	73.58	±3.57 ^c^	255.61	±10.15 ^b^	22.94	±1.34 ^a^	99.01 ^d^
Rustic	54.28	±2.61 ^b^	196.52	±9.71 ^a^	325.11	±15.96 ^a,b^	24.11	±1.25 ^a^	250.8 ^b^
Tampa	34.81	±1.69 ^c^	167.92	±8.29 ^b^	350.29	±17.33 ^a^	22.56	±1.33 ^a^	202.73 ^c^
Zamolxis	24.03	±1.21 ^c^	52.78	±2.54 ^c^	238.41	±11.83 ^b^	23.39	±1.35 ^a^	76.81 ^d^
Min	24.03		52.78		238.41		18.74		76.81
Max	76.58		232.97		378.45		24.11		309.55

**Table 2:** Correlation matrix for the studied variables.

	Fructose	Glucose	Sucrose	DM
Fructose	1			
Glucose	0.8748	1		
Sucrose	0.2933	0.6527	1	
DM	−0.4622	−0.1181	0.4013	1

Note: DM—dry matter.

**Table 3:** Mean values of SCC in potato cultivars at harvest expressed in g/100 g DW (means followed by different superscript letters within the same column indicate significant differences, established using one-way ANOVA with post-hoc Tukey’s HSD test (*p* < 0.05)).

Cultivar	Fructose	Glucose	Sucrose	Reducing Carbohydrates	Total SCC
Christian	0.17 ^c^	0.30 ^b^	1.29 ^b^	0.47 ^c^	1.76 ^c^
Cumidava	0.16 ^c^	0.64 ^a^	1.57 ^a^	0.80 ^b^	2.38 ^b^
Kronstadt	0.12 ^c^	0.30 ^b^	1.17 ^b,c^	0.42 ^c^	1.59 ^c^
Riviera	0.41 ^a^	1.24 ^b^	1.51 ^a^	1.65 ^a^	3.17 ^a^
Roclas	0.11 ^c^	0.32 ^b^	1.12 ^c^	0.43 ^c^	1.55 ^c^
Rustic	0.23 ^b^	0.82 ^a^	1.35 ^a,b^	1.04 ^b^	2.39 ^b^
Tampa	0.15 ^c^	0.74 ^a^	1.55 ^a^	0.90 ^b^	2.45 ^b^
Zamolxis	0.10 ^c^	0.23 ^b^	1.02 ^c^	0.33 ^c^	1.35 ^c^
Min	0.10	0.23	1.02	0.33	1.35
Max	0.41	1.24	1.57	1.65	3.17

**Table 4:** Calibration details.

Carbohydrate	Calibration Range [mg/L)	Equation	R^2^
Fructose	22.04–551.03	C = 0.012827 × A + 0.693289	0.99756
Glucose	16.97–424.31	C = 0.014052 × A + 2.871871	0.99893
Sucrose	16.22–911.52	C = 0.024985 × A + 2.252326	0.99581

Note: C—concentration; A—peak area.

## Data Availability

Not applicable.
